# Ill Fate of Rectal Mucinous Adenocarcinoma: A Defect in Immunosurveillance or a Mucin Coating Effect?—The IMMUNOREACT 20 Study

**DOI:** 10.3390/cancers18121943

**Published:** 2026-06-15

**Authors:** Lorenzo Dell’Atti, Andromachi Kotsafti, Francesca Galuppini, Melania Scarpa, Roberta Salmaso, Astghik Stepanyan, Marta Sbaraglia, Luca Maria Saadeh, Gaia Tussardi, Antonio Rosato, Imerio Angriman, Cesare Ruffolo, Emanuele Damiano Luca Urso, Quoc Riccardo Bao, Silvia Negro, Isacco Maretto, Luca Facci, Giorgio Rivella, Antonella D’Angelo, Anna Matteazzi, Chiara Vignotto, Andrea Baldo, Vincenza Guzzardo, Valerio Pellegrini, Stefano Brignola, Carlotta Ceccon, Tommaso Stecca, Anna Pozza, Marco Massani, Ottavia De Simoni, Pierluigi Pilati, Mario Gruppo, Boris Franzato, Ivana Cataldo, Giuseppe Portale, Chiara Cipollari, Matteo Zuin, Licia Laurino, Luca Dal Santo, Giovanni Pirozzolo, Alfonso Recordare, Lavinia Ceccarini, Michele Antoniutti, Laura Marinelli, Alberto Brolese, Mattia Barbareschi, Giovanni Bertalot, Monica Ortenzi, Mario Guerrieri, Maurizio Zizzo, Massimiliano Fabozzi, Silvio Guerriero, Alessandra Piccioli, Giulia Pozza, Mario Godina, Isabella Mondi, Daunia Verdi, Corrado Da Lio, Giulia Noaro, Roberto Cola, Giovanni Bordignon, Roberto Merenda, Giulia Becherucci, Laura Gavagna, Salvatore Candioli, Giovanni Tagliente, Umberto Tedeschi, Dario Parini, Beatrice Salmaso, Gianluca Businello, Loretta Di Cristofaro, Francesco Marchegiani, Francesca Bergamo, Sara Lonardi, Andrea Porzionato, Valentina Chiminazzo, Federico Scognamiglio, Romeo Bardini, Salvatore Pucciarelli, Marco Agostini, Dario Gregori, Barbara Di Camillo, Ignazio Castagliuolo, Gaya Spolverato, Matteo Fassan, Angelo Paolo Dei Tos, Marco Scarpa

**Affiliations:** 1 Surgical Oncology Unit, Azienda USL-IRCCS di Reggio Emilia, 42122 Reggio Emilia, Italy; lorenzo.dellatti@ausl.re.it (L.D.);; 2 Veneto Institute of Oncology IOV-IRCCS, 35128 Padova, Italy; 3 Department of Integrated Diagnostics, Azienda Ospedale-Università Padova, 35128 Padova, Italy; 4 Department of Medicine, University of Padova School of Medicine, 35128 Padova, Italy; 5 Chirurgia Generale 3, Azienda Ospedale Università di Padova, 35131 Padova, Italy; 6 Dipartimento di Scienze Chirurgiche, Oncologiche e Gastroenterologiche (DISCOG), University of Padova, 35128 Padova, Italy; 7 Pathology Unit, Ospedale Ca’Foncello, AULSS 2 Marca Trevigiana, 31100 Treviso, Italy; 8 General Surgery Unit 3, Ospedale Ca’ Foncello, AULSS 2 Marca Trevigiana, 31100 Treviso, Italy; 9 Surgical Oncology of Digestive Tract Unit, Veneto Institute of Oncology IOV-IRCCS, 35128 Padova, Italy; 10 Oncological Anatomy and Histology Unit, Veneto Institute of Oncology IOV-IRCCS, 35128 Padova, Italy; 11 General Surgery Unit, Ospedale Alto Vicentino, Azienda ULSS 7 Pedemontana, 36014 Santorso, Italy; 12 General Surgery Unit, Ospedale di Cittadella, Azienda ULSS 6 Euganea, 35013 Cittadella, Italy; 13 Pathology Unit, Ospedale Dell’Angelo, Azienda ULSS 3 Serenissima, 30174 Venezia, Italy; 14 General Surgery Unit, Ospedale San Bassiano, Azienda ULSS 7 Pedemontana, 36061 Bassano del Grappa, Italy; 15 Department of General Surgery & HPB Unit, ASUIT—Azienda Sanitaria Universitaria Integrata del Trentino, 38122 Trento, Italy; 16 Pathology Unit, Ospedale Santa Chiara, CISMed University of Trento c/o ASUIT, 38122 Trento, Italy; 17 General Surgery Unit, Ospedali Riuniti, 60126 Ancona, Italy; 18 General Surgery Unit, Ospedale di Fermo, ASUR 4, 63900 Fermo, Italy; 19 Bariatric Surgery Unit, Azienda Ospedale Università di Padova, 35128 Padova, Italy; 20 General Surgery Unit, Ospedale di Dolo, Azienda ULSS 3, 30031 Venezia, Italy; 21 General Surgery Unit, Ospedale di Mirano, Azienda ULSS 3 Serenissima, 30035 Venezia, Italy; 22 General Surgery Unit, Ospedali Riuniti Padova Sud Madre Teresa di Calcutta, Azienda ULSS 6 Euganea, 35043 Padova, Italy; 23 General Surgery Unit, Ospedale Santi Giovanni e Paolo, Azienda ULSS 3 Serenissima, 30122 Venezia, Italy; 24 General Surgery Unit, Ospedale San Martino, Azienda ULSS 1 Dolomiti, 32100 Belluno, Italy; 25 General Surgery Unit, Ospedale Dell’Angelo, Azienda ULSS 3 Serenissima, 30174 Venezia, Italy; 26 General Surgery Unit, Casa di Cura di Abano, 35031 Padova, Italy; 27 General Surgery Unit, Ospedale Santa Maria della Misericordia, AULSS 5 Polesana, 45100 Rovigo, Italy; 28 Pathology Unit, Ospedale Santa Maria della Misericordia, AULSS 5 Polesana, 45100 Rovigo, Italy; 29 General Surgery Unit, Ospedale Grassi, ASL ROMA 3, 00122 Ostia, Italy; 30 Unit of Colorectal and Digestive Surgery, Beaujon Hospital, 92110 Paris, France; 31 Medical Oncology 1, Veneto Institute of Oncology IOV-IRCCS, University of Padova, 35128 Padova, Italy; 32 Department of Neurosciences (DNS), University of Padova, 35128 Padova, Italy; 33 Health Research Institute of the Principality of Asturias (ISPA), 33011 Oviedo, Spain; 34 Department of Cardiac, Thoracic, Vascular Sciences and Public Health, University of Padova, 35128 Padova, Italy; 35 Information Engineering (DEI), University of Padova, 35128 Padova, Italy; 36 Department of Molecular Medicine (DMM), University of Padova, 35128 Padova, Italy

**Keywords:** rectal cancer, mucinous adenocarcinoma, tumour microenvironment, immunosurveillance, field cancerisation, propensity score matching, immune evasion, colorectal cancer

## Abstract

Background/Objectives: Mucinous adenocarcinoma (MAC) is a rare and clinically problematic subtype of rectal cancer, tending to present at an advanced stage and to respond poorly to neoadjuvant therapy. The consistently worse prognosis than that of not-otherwise-specified adenocarcinoma (NOS-AC) is not fully understood, potentially owing to intrinsically more aggressive biology or specific immune evasion mechanisms. We used the IMMUNOREACT multicentre cohort, with external validation in TCGA, to investigate the clinical and immunological features of rectal MAC in detail. Methods: Two hundred patients with rectal adenocarcinoma (16 MAC, 184 NOS-AC) from the IMMUNOREACT 1 (NCT04915326) and IMMUNOREACT 2 (NCT04917263) prospective cohorts were included. To account for the imbalance in baseline characteristics, propensity score matching (PSM) was performed on age, sex, neoadjuvant treatment and TNM stage. The immune microenvironment was characterised using immunohistochemistry (CD3, CD4, CD8, CD8β, Tbet, FoxP3, PD-L1, MSH6, PMS2, CD80), flow cytometry and NanoString PanCancer IO 360™ transcriptomics of adjacent healthy mucosa. Findings were externally validated against TCGA rectal and colon adenocarcinoma datasets. Results: MAC presented at significantly more advanced stage than NOS-AC across all TNM parameters: higher T stage (*p* = 0.006), N stage (*p* < 0.001), M stage (*p* = 0.039) and overall TNM stage (*p* < 0.001). In the unmatched cohort, MAC was associated with worse overall survival (HR 2.53; 95% CI 1.03–6.23; *p* = 0.043) and disease-free survival (HR 2.86; 95% CI 1.25–6.55; *p* = 0.013), but both differences became non-significant after PSM. MAC patients had higher haemoglobin after adjusting for confounders (mean difference [MD] 1.26 g/dL, 95% CI 0.30–2.31, *p* = 0.012), consistent with a hypothesis of reduced chronic rectal bleeding as a possible mechanism for late presentation. Transcriptomically, MAC showed suppression of HLA class II antigen presentation genes (HLA-DQA1, HLA-DQB1, HLA-DRB1) and myeloid activation genes (S100A8/A9/A12) in adjacent healthy mucosa. Loss of MMR proteins MSH6 and PMS2 in histologically normal mucosa was significantly more frequent in MAC. These findings were replicated in the TCGA cohort, which also showed lower tumour mutational burden and a distinct mucin-associated transcriptomic profile in MAC. Conclusions: The worse outcomes of rectal MAC appear to be driven largely by late-stage presentation, possibly owing to later diagnosis. MAC nonetheless carries a distinct immune phenotype, detectable even in histologically normal surrounding mucosa, that likely contributes to its treatment resistance. These observations provide a basis for developing histotype-specific approaches to both early detection and treatment in this uncommon but clinically challenging tumour subtype.

## Simple Summary

Mucinous adenocarcinoma is a rare subtype of rectal cancer that tends to respond poorly to standard treatments, leading to worse outcomes than typical rectal cancer. The underlying reasons are not fully understood. Using a multicentre cohort of 200 patients, we show that the survival disadvantage largely disappears once the difference in disease stage at diagnosis is accounted for, suggesting that, rather than more aggressive tumour biology, late detection may be the main driver of poor outcomes. Higher haemoglobin levels in mucinous adenocarcinoma patients may point to the possibility that these tumours bleed less, delaying the alarm symptoms that usually prompt investigation. Molecular analysis of the tissue surrounding the tumour reveals a distinct immune fingerprint, with reduced expression of genes involved in immune recognition, even in histologically normal mucosa. These findings point toward new strategies for earlier detection and immune-based treatment of this uncommon but challenging cancer subtype.

## 1. Introduction

Colorectal cancer (CRC) is the third most commonly diagnosed malignancy worldwide and the second leading cause of cancer death [1]. While age-adjusted incidence has been falling in older adults, early-onset disease has risen steadily across a number of countries over the past two decades [2]. The vast majority of rectal cancers are adenocarcinomas of the not-otherwise-specified type (NOS-AC), which account for 85–90% of cases. Mucinous adenocarcinoma (MAC) is a morphologically distinct subtype, defined by the WHO as tumours in which extracellular mucin pools make up more than 50% of the lesion [3]. In the rectum this histotype is relatively uncommon, representing roughly 4.6% of cases [4].

What sets MAC apart clinically is a tendency to present at an advanced stage. Compared to NOS-AC, MAC tumours are more likely to be T4 at diagnosis, show greater nodal involvement and have a larger primary size [4]. There is also a well-documented resistance to neoadjuvant chemoradiotherapy, attributed to several overlapping mechanisms: the dense mucin matrix physically impedes drug penetration, altered expression of chemotherapy-metabolising enzymes reduces drug efficacy and mucin glycoproteins appear to confer intrinsic anti-apoptotic properties [5]. Taken as a whole, these features translate into lower rates of pathological downstaging and reduced long-term survival. An additional layer of complexity is introduced by the relationship between mucinous histology and mismatch repair (MMR) status. In colonic MAC approximately 29–42% of tumours are microsatellite instability-high (MSI-high), compared to around 15% of NOS-AC; in the rectum, however, most MAC is microsatellite stable (MSS), with MSI-H rates of only 12–13% [6,7].

Despite this fairly well-characterised clinical profile, relatively little is known about the immune biology of rectal MAC. Mucin glycoproteins can promote an immunosuppressive microenvironment through adhesion ligand expression and cytokine secretion [8], but a systematic characterisation of the immune infiltrate, antigen presentation machinery and co-stimulatory pathways in this histotype has not been carried out [9]. A multiplexed immunofluorescence study of mucinous rectal cancer did find an immune-rich microenvironment with elevated lymphocyte infiltration and enhanced PD-1 expression, independent of MSI status [10], suggesting that MAC is not immune-cold but rather immune-infiltrated yet functionally impaired. Working within the IMMUNOREACT multicentre cohort, we aimed to define the clinical features and immune microenvironment of rectal MAC and, in doing so, explore whether the poor prognosis of this subtype reflects a stage effect, an intrinsically more aggressive biology or some combination of immune evasion mechanisms.

## 2. Materials and Methods

### 2.1. Study Design

**Figure 1:**
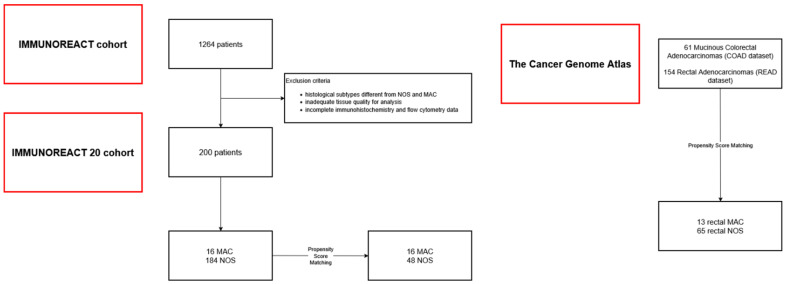
Study flowchart. IMMUNOREACT 1 (NCT04915326) enrolled patients with early rectal carcinoma; IMMUNOREACT 2 (NCT04917263) enrolled patients undergoing neoadjuvant therapy for locally advanced rectal tumour. MAC = mucinous adenocarcinoma; NOS-AC = not-otherwise-specified adenocarcinoma; PSM = propensity score matching; TCGA = The Cancer Genome Atlas.

The IMMUNOREACT project is a multicentre observational study investigating immunosurveillance mechanisms in rectal cancer through immunohistochemistry and gene expression profiling in the adjacent healthy mucosa. This study is a retrospective analysis of clinical, histopathological and immunophenotypical data from two complementary prospective multicentre cohorts within the IMMUNOREACT framework: IMMUNOREACT 1, enrolling early rectal carcinoma patients (ClinicalTrials.gov NCT04915326), and IMMUNOREACT 2, enrolling patients who underwent neoadjuvant therapy for locally advanced rectal tumour (NCT04917263). Additional data were obtained from The Cancer Genome Atlas (TCGA; https://www.cancer.gov/tcga, accessed on 1 November 2025). The study design is illustrated in Figure 1.

### 2.2. Ethical Approval

The IMMUNOREACT protocol received ethical approval from the Institutional Review Board of the coordinating centre (Padova University Hospital; Protocol #4448/AO/20) and from the ethics committees of all participating institutions. Written informed consent was obtained from all enrolled participants. The study was conducted in accordance with the Declaration of Helsinki.

### 2.3. Patients and Eligibility Criteria

MAC was defined per WHO 2010 criteria as adenocarcinoma in which extracellular mucin comprises ≥50% of the tumour volume [11]. Data were collected anonymously using the REDCap platform [12]. Clinical staging followed TNM 8th edition criteria [13]. Inclusion criteria were: histologically confirmed rectal adenocarcinoma (MAC or NOS-AC), age ≥ 18 years, availability of rectal mucosal tissue and complete immunohistochemistry data. Exclusion criteria were: other histological subtypes (signet ring cell, adenosquamous, mixed adenoneuroendocrine or serrated adenocarcinomas) and inadequate tissue quality.

### 2.4. Tissue Sampling and Immunohistochemistry

During surgical resection, biopsies of macroscopically normal rectal mucosa were harvested at 3–15 cm proximal to the primary tumour margin, consistent with the field cancerisation framework [14,15]. All samples were subject to histopathological review by a board-certified gastrointestinal pathologist who confirmed preserved crypt architecture and the absence of high-grade dysplasia and active inflammation prior to inclusion in downstream analyses; this review was performed blinded to histotype, following the standard operating procedure applied across the IMMUNOREACT project. Immunohistochemical (IHC) staining was performed on formalin-fixed paraffin-embedded (FFPE) tissue microarrays (TMAs) cut at 5 µm. Signal detection employed an avidin–biotin–peroxidase conjugate with 3,3’-diaminobenzidine tetrahydrochloride chromogen (Dako, Glostrup, Denmark). Slides were scored by a single pathologist blinded to all clinical data; the first 50 cases were independently co-scored by two pathologists (M.F. and V.P.), yielding high inter-rater agreement (Cohen’s κ > 0.85). The marker panel comprised CD3, CD4, CD8, CD8β, Tbet, FoxP3, PD-L1, MSH6, PMS2 and CD80. Mismatch repair (MMR) protein status was assessed using a two-antibody approach (MSH6 and PMS2) that demonstrates strong agreement with four-antibody panels in colorectal adenocarcinoma [16,17].

### 2.5. Flow Cytometry

Fresh rectal mucosa samples collected prospectively were enzymatically digested into single-cell suspensions and labelled with FITC-, PE-, PE-Cy7-, and APC-conjugated antibodies. The following cell populations were characterised: epithelial cells (pan-cytokeratin+) co-expressing CD80, CD86 and HLA-ABC; activated cytotoxic T cells (CD8+CD28+CD38+); inhibitory T cells (CD3+CTLA-4+); activated T helper cells (CD4+CD25+); and regulatory T cells (CD4+CD25+FoxP3+). Acquisition and analysis were performed on a FACSCalibur instrument with CellQuest software version 5.1 (BD Biosciences, Franklin Lakes, NJ, USA).

### 2.6. Gene Expression Profiling

Total RNA was extracted from snap-frozen rectal mucosa using the RNeasy Plus Mini Kit (Qiagen, Germantown, MD, USA). RNA concentration was quantified using the ND-1000 UV–VIS Spectrophotometer (NanoDrop Technologies, Wilmington, DE, USA), and RNA integrity was assessed using the Agilent 4200 TapeStation and RNA ScreenTape Assay (Agilent Technologies, Santa Clara, CA, USA). Gene expression profiling was performed using the PanCancer IO 360™ panel (NanoString Technologies, Seattle, WA, USA), which measures 770 genes involved in tumour–immune interactions. For hybridisation, 300 ng of total RNA per sample was incubated overnight at 65 °C with fluorescently barcoded reporter probes and biotin-labelled capture probes, according to the manufacturer’s instructions. Hybridised complexes were immobilised and digitally counted using the NanoString nCounter Digital Analyzer, which quantifies transcript abundance through barcode detection. Raw data underwent quality control and normalisation using nSolver Analysis Software v4.0 (NanoString Technologies). For the MAC group, profiling was performed in the four patients for whom adequate snap-frozen tissue was available; the NOS-AC comparison group (*n* = 12) was selected based on matched stage at presentation. Differential expression analysis was performed using the NanoString nCounter Advanced Analysis module within nSolver Analysis Software v4.0 (NanoString Technologies, Seattle, WA, USA). *p*-values were adjusted for multiple testing using the Benjamini–Yekutieli (BY) correction method. Given the limited sample size, transcriptomic findings were considered exploratory and hypothesis-generating. Differential expression results, including log2 fold change, raw *p*-values and BY-adjusted *p*-values for all 770 analysed genes, are reported in Supplementary Table S1. GraphPad Prism 10.0.0 was used for graphical representation of normalised gene expression data.

### 2.7. Mutational Analysis

Targeted next-generation sequencing (NGS) was performed on FFPE samples of adjacent histologically normal mucosa using the Archer^®^ VariantPlex^®^ Solid Tumour Panel (ArcherDX, Boulder, CO, USA), enabling detection of single-nucleotide variants in 63 genes and copy number variation in 44 genes. Libraries were sequenced on a NextSeq 550 platform (Illumina, San Diego, CA, USA) using paired-end 2 × 151 bp reads. Sequencing data were aligned to the hg19 (GRCh37) human reference genome and analysed using Archer^®^ Analysis v6.0 software. Variant calling was performed using default software parameters; no pre-specified minimum depth threshold beyond the software default (minimum 4× coverage) was applied, and the minimum variant allele frequency (VAF) threshold was 0.001%, consistent with the software default. The lowest VAF observed among retained variants in this dataset was 2%. Common population polymorphisms were filtered using gnomAD and ClinVar databases. The mutational ratio was defined as the number of genes in which at least one variant was detected divided by the total number of genes with adequate sequencing coverage per sample. Because sequencing was carried out on histologically normal mucosa rather than tumour, the variants detected reflect the constitutive and field-level landscape of each patient rather than tumour-specific somatic mutations. For this reason, and because the assay interrogates a targeted panel of recurrently mutated genes rather than the whole exome, the mutational ratio should be considered as a surrogate measure of the field mutational landscape and should not be equated with tumour mutational burden as defined by whole-exome analyses.

### 2.8. TCGA Cohort

Molecular data were obtained from the TCGA rectum adenocarcinoma (READ) and colon adenocarcinoma (COAD) datasets. A total of 61 mucinous colorectal adenocarcinoma cases were compared with 155 non-mucinous rectal adenocarcinoma cases; 13 mucinous rectal carcinomas were further compared to 65 propensity-score-matched non-mucinous rectal adenocarcinomas.

### 2.9. Statistical Analysis

Continuous variables are reported as median (IQR), and categorical variables as frequency (%). Group comparisons used the Mann–Whitney test, Chi-squared test, or Fisher’s exact test as appropriate. Correlations were assessed with Kendall’s tau. Multivariable linear regression evaluated the independent association of histological subtype with haemoglobin levels. Overall survival (OS) was defined as time from surgery to death from any cause, and disease-free survival (DFS) as time to first recurrence or cancer-related death. Survival curves were estimated by the Kaplan–Meier method and compared with the log-rank test; effect sizes are expressed as hazard ratio (HR) with 95% confidence interval (CI). Propensity score matching (PSM) was performed using 1:3 nearest-neighbour matching based on age, sex, neoadjuvant treatment and TNM stage. All tests were two-tailed at α = 0.05. Analyses were performed using GraphPad Prism 10.0.0 (GraphPad Software, Boston, MA, USA).

## 3. Results

### 3.1. Patient Characteristics and Oncologic Outcomes

**Figure 2:**
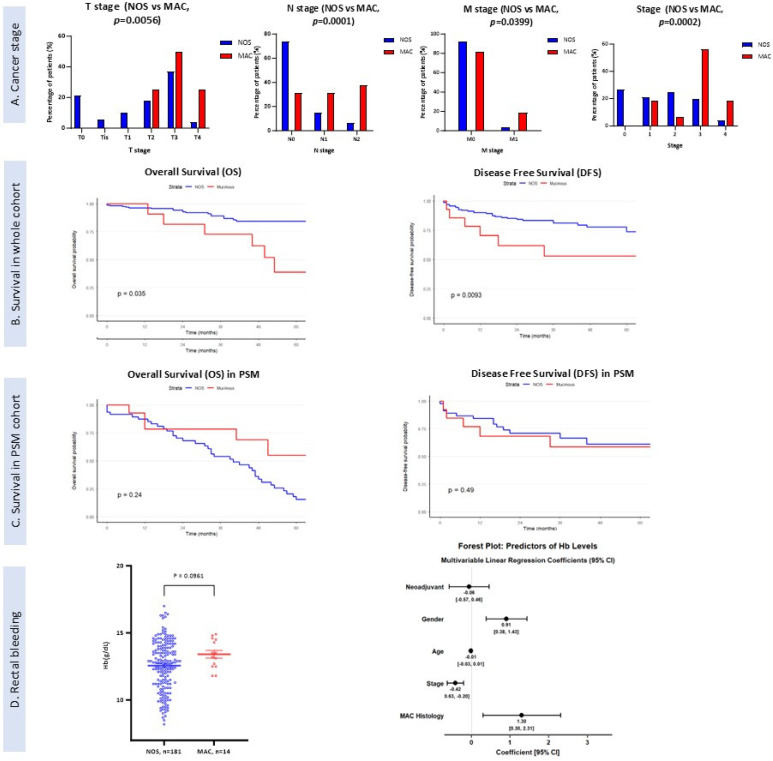
Stage at presentation and survival outcomes in the IMMUNOREACT 20 cohort. (**A**) Pathological T, N, M, and overall TNM stage distribution in the whole cohort. (**B**) Kaplan–Meier curves for OS and DFS in the whole cohort. (**C**) Kaplan–Meier curves for OS and DFS in the PSM cohort. (**D**) Haemoglobin levels and multivariable linear regression analysis. MAC = mucinous adenocarcinoma; NOS-AC = not-otherwise-specified adenocarcinoma; HR = hazard ratio; CI = confidence interval; MD = mean difference; PSM = propensity score matching.

A total of 200 patients were included: 16 (8%) with MAC and 184 (92%) with NOS-AC (Table 1). Demographics were comparable (age: 59 [IQR 12.5] vs. 66 [IQR 21] years, *p* = 0.171; male sex: 68.8% vs. 57.6%, *p* = 0.195). Despite comparable demographics, MAC presented at significantly more advanced pathological stages: higher T stage (*p* = 0.006), N stage (*p* < 0.001), M stage (*p* = 0.039) and overall TNM stage (*p* < 0.001). Notably, 25% of MAC patients presented with T4 tumours (vs. 2.6%), 37.5% had N2 disease (vs. 6.3%) and 18.8% had distant metastases (vs. 3.8%). No MAC patient presented at stage 0 (vs. 27.4% of NOS-AC; Figure 2A). After PSM, the matched cohort comprised 64 patients (16 MAC, 48 NOS-AC), with balanced baseline characteristics (Supplementary Figure S1A).

### 3.2. Haemoglobin Levels as a Potential Diagnostic Clue

To explore reasons for the delayed MAC presentation, we analysed haemoglobin (Hb) as a surrogate of chronic rectal bleeding. MAC patients had higher Hb levels (13.4 [SD 1.08] vs. 12.6 [SD 1.90] g/dL; *p* = 0.096), a difference that reached significance after multivariable adjustment for stage, age, sex and neoadjuvant therapy (whole cohort: mean difference [MD] 1.26 g/dL, 95% CI 0.30–2.31, *p* = 0.012; PSM cohort: MD 1.13, 95% CI 0.01–2.16, *p* = 0.043; Figure 2D). This finding is consistent with the hypothesis that MAC may bleed less, thereby delaying symptom onset and contributing to late-stage presentation; however, no prospective data on presenting symptoms were collected and haemoglobin has to be considered an indirect and imperfect surrogate for rectal bleeding.

### 3.3. Mutational Analysis

**Figure 3:**
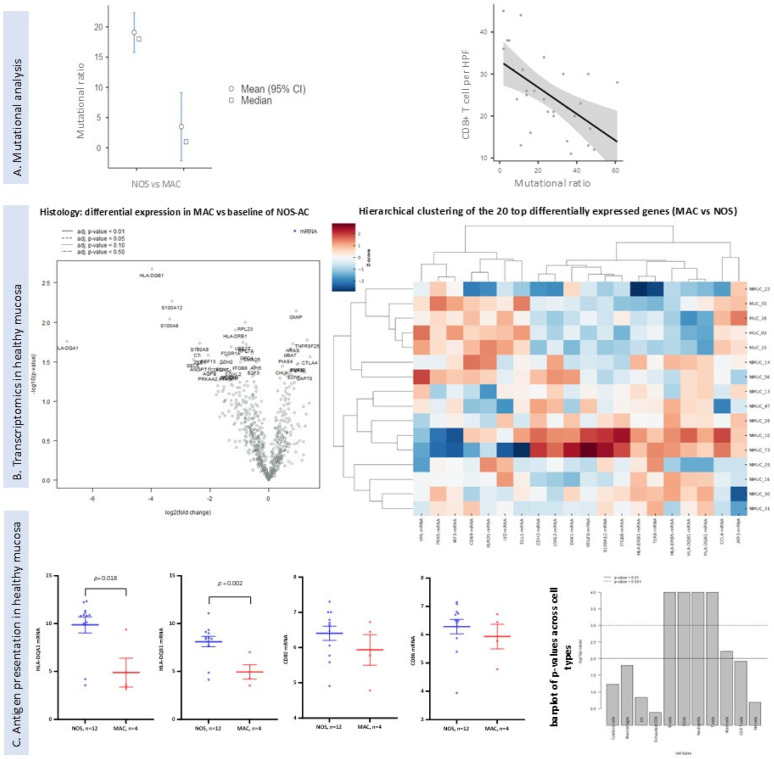
Mutational and transcriptomic analyses. (**A**) Mutational ratio in MAC vs. NOS-AC (left) and scatter plot of inverse correlation with CD8+ T-cell density (right). (**B**) Volcano plot (left) and hierarchical clustering heatmap of top 20 differentially expressed genes (right); z-scores: red +2, white 0, blue −2. (**C**) HLA-DQA1, HLA-DQB1, CD80, and CD86 mRNA expression (left) and cell-type infiltrate proportions (right). MAC = mucinous adenocarcinoma; NOS-AC = not-otherwise-specified adenocarcinoma.

In the IMMUNOREACT series, there were no notable differences between the MAC and NOS-AC groups in terms of single gene mutation (Supplementary Table S2). However, MAC patients had a median of 4 (IQR 3) mutated genes compared to 11 (IQR 9) in NOS-AC (*p* = 0.009). The mutational ratio (total mutated genes divided by total genes successfully sequenced) was significantly lower in MAC than in NOS-AC patients (*p* = 0.028; Figure 3A). Furthermore, CD8+ T-cell density was inversely correlated with mutational burden (Kendall tau = −0.347, *p* = 0.002).

### 3.4. Transcriptomic Analysis

Due to limited tissue availability, gene expression profiling was carried out in 4 MAC patients and 12 NOS-AC patients. MAC demonstrated suppression of antigen presentation machinery with downregulation of HLA class II genes: HLA-DQA1 (*p* = 0.008), HLA-DQB1 (*p* = 0.02) and HLA-DRB1 (*p* = 0.013). MAC additionally showed suppression of myeloid-compartment genes including S100A12 (*p* = 0.005), S100A8 (*p* = 0.009) and S100A9 (*p* = 0.019; Figure 3B) [18]. Conversely, upregulation of GMIP [19] (*p* = 0.007) and TNFRSF25 (*p* = 0.017) suggested dysregulated costimulatory and death receptor signalling. Given that no genes survived Benjamini–Yekutieli correction across the full 770-gene panel, these *p*-values are nominal and the findings must be interpreted cautiously (Supplementary Table S1). Hierarchical clustering of the top 20 differentially expressed genes tended to separate the two histotypes (Figure 3B). Cell-type profiling suggested differences in B-cell, T-cell, neutrophil and mast cell infiltration (all *p* ≤ 0.01; Figure 3C), with MAC showing higher overall B- and T-cell infiltration despite suppressed antigen presentation machinery. However, given the sample size of four MAC cases, these findings should be regarded as exploratory and hypothesis-generating.

### 3.5. Immunohistochemistry and Flow Cytometry

**Figure 4:**
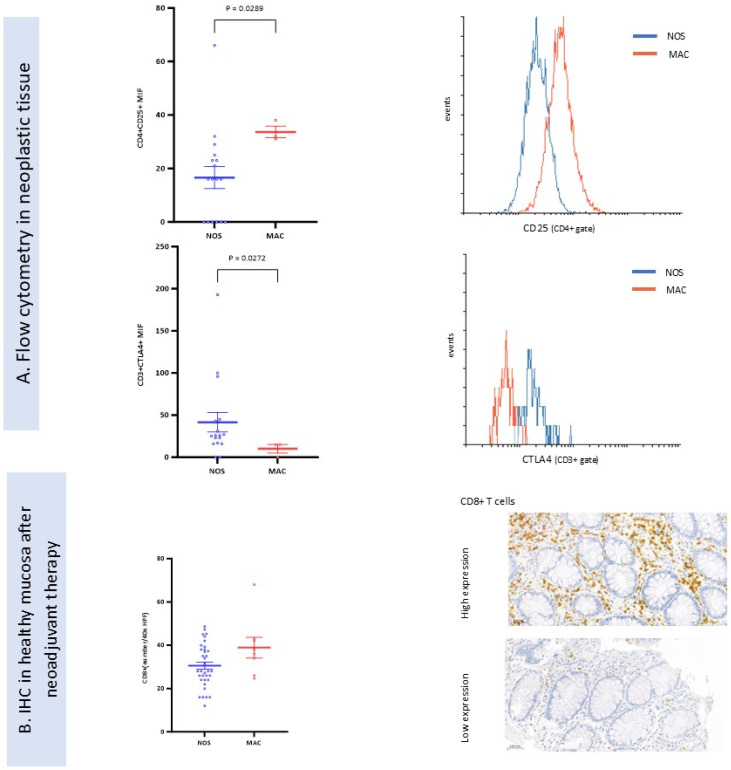
Immunohistochemistry and flow cytometry results. (**A**) Flow cytometry: proportions of CD4+CD25+ and CD3+CTLA-4+ in MAC vs. NOS-AC neoplastic tissue. (**B**) CD8+ infiltration in adjacent healthy mucosa in patients receiving neoadjuvant therapy. MAC = mucinous adenocarcinoma; NOS-AC = not-otherwise-specified adenocarcinoma.

In neoplastic tissue, MAC showed elevated CD4+CD25+ cell proportions (*p* = 0.027; Figure 4A), suggesting increased but potentially dysfunctional CD4+ T-cell activation. CD3+CTLA-4+ lymphocytes were significantly lower in MAC (*p* = 0.034; Figure 4A). In adjacent healthy mucosa, loss of MSH6 and PMS2 expression was observed more frequently in MAC (MSH6 loss: 8.3% vs. 0%, *p* = 0.036; PMS2 loss: 16.7% vs. 0%, *p* = 0.004), a finding that warrants further independent validation. Among patients who received neoadjuvant therapy (*n* = 108), PMS2 loss was more frequent in MAC (28.6% vs. 0%, *p* = 0.007) and CD8+ lymphocyte infiltration in healthy mucosa was higher in MAC (*p* = 0.049; Figure 4B). In the PSM cohort, IHC markers did not differ significantly, while flow cytometry confirmed elevated CD4+CD25+ cells in MAC neoplastic tissue (*p* = 0.04; Supplementary Figure S1B,C). Microsatellite instability status was available for a subset of patients (16 MAC and 100 NOS-AC) and did not differ significantly between histotypes, with an MSI-high pattern in 1 of 16 MAC (6.25%) and 8 of 100 NOS-AC (8.0%) cases (*p* = 0.808).

### 3.6. TCGA Cohort Validation

**Figure 5:**
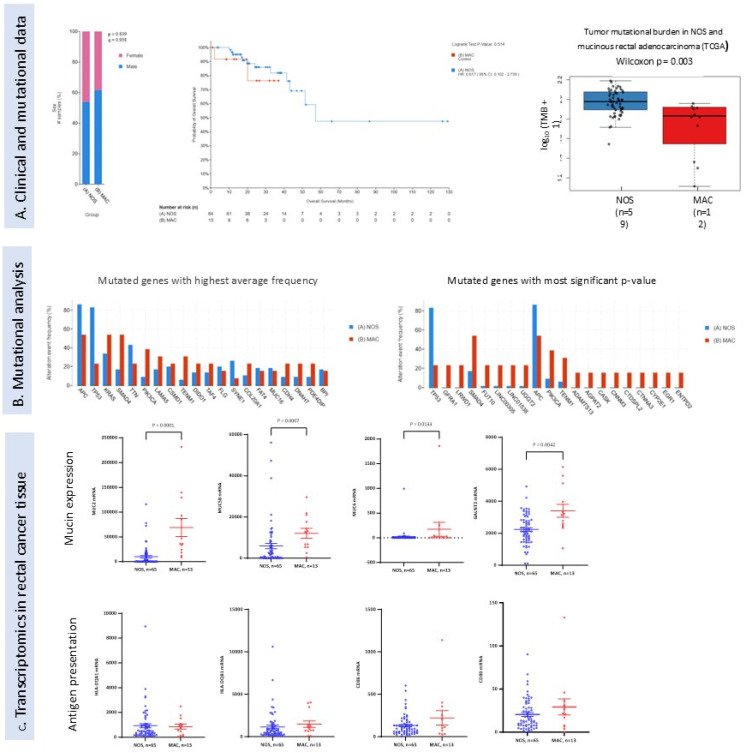
TCGA cohort results (13 MAC vs. 65 PSM-matched NOS-AC rectal adenocarcinomas). (**A**) Sex distribution. (**B**) Overall survival Kaplan–Meier curves. (**C**) Mutated gene distribution. MAC = mucinous adenocarcinoma; NOS-AC = not-otherwise-specified adenocarcinoma; TCGA = The Cancer Genome Atlas.

Analysis of the TCGA rectal cohort confirmed no significant OS difference between 13 MAC and 65 matched NOS-AC patients (HR 0.617; 95% CI 0.102–3.739; *p* = 0.091), consistent with our PSM findings (Table 2; Figure 5A,B).

## 4. Discussion

The main take-home message from IMMUNOREACT 20 is that the poor prognosis associated with rectal MAC may be largely a staging artefact. In the unmatched cohort, MAC carried a more than 2.5-fold excess risk of death and recurrence. Once we adjusted for stage, age, sex and neoadjuvant therapy through propensity score matching, that disadvantage disappeared. The survival gap is driven principally by the fact that MAC patients present at a much later stage.

The reason for this later presentation may be found in our haemoglobin analysis. MAC patients had higher Hb values than NOS-AC patients; this result persisted after adjusting for stage and other confounders (whole cohort MD 1.26 g/dL, 95% CI 0.30–2.31, *p* = 0.012). Rectal bleeding is the dominant alarm symptom that brings rectal cancer to clinical attention. If MAC is less prone to bleeding it is plausible that a proportion of these patients never develop the symptom that would trigger endoscopic investigation. Instead they present later, with obstruction or a vague change in bowel habit, by which time the disease is already advanced. This is a speculative hypothesis, since no prospective data on presenting symptoms were collected and haemoglobin is an indirect surrogate influenced by multiple factors beyond bleeding alone. A prospective study capturing rectal bleeding as a clinical variable would be needed to confirm or refute this interpretation, but the haemoglobin finding provides a biological substrate that makes the hypothesis worth testing.

The translational analyses paint a picture of a tumour that evades immune recognition from the outset. In the adjacent normal mucosa, MAC showed a markedly lower mutational ratio than NOS-AC and CD8+ T-cell density was inversely correlated with this field-level mutational load (Kendall tau = −0.347, *p* = 0.002). However, this result represents the mutational landscape of the cancerisation field, which encompasses both germline variants and somatic alterations acquired through field exposure, rather than tumour-specific somatic burden. A field carrying fewer such alterations would nonetheless be expected to give rise to a tumour with fewer somatic mutations, and therefore fewer neoantigens. This expectation is further confirmed in the TCGA data, where MAC tumours showed a lower tumour mutational burden than NOS-AC, consistent with a weaker neoantigen-specific T-cell response and, as a consequence, reduced cytotoxic infiltration [18].

According to the concept of field cancerisation [14,15], we performed transcriptomic analysis of histologically normal mucosa to further investigate potential differences. In MAC, the adjacent mucosa showed coordinated downregulation of HLA class II genes (HLA-DQA1, HLA-DQB1, HLA-DRB1) and the S100 family (S100A8, S100A9, S100A12). The S100 proteins are damage-associated molecular patterns (DAMPs) with a role in recruiting and activating myeloid cells [18]; recent work in colorectal cancer has confirmed that S100A8 and S100A9 in particular drive the recruitment and differentiation of myeloid-derived suppressor cells (MDSCs) and M2-polarised macrophages within the tumour microenvironment [20]. Their coordinated suppression in MAC adjacent mucosa may therefore reflect a broader failure of innate immune alarm signalling, reducing the myeloid infiltration that would otherwise flag early tissue damage. The HLA class II downregulation in phenotypically normal tissue suggests that the defect in CD4+ T-cell priming begins well before a recognisable tumour is established. A recent proteogenomic study of HLA-II-negative colorectal cancer showed that neoantigen-specific CD4+ surveillance can still occur via stromal antigen-presenting cells (APCs) and that the density of such stromal APCs correlates positively with tumour mutational burden [21]. Whether the HLA class II downregulation we observe is cause or consequence of the mucinous phenotype cannot be determined from cross-sectional data, but it is a provocative finding.

The IHC and flow cytometry results add further nuance. MAC neoplastic tissue contained more CD4+CD25+ cells than NOS-AC, yet fewer CD3+CTLA-4+ lymphocytes. An elevated CD4+CD25+ fraction might suggest immune activation, while reduced CTLA-4 expression would imply less engagement of the inhibitory checkpoint arm. Importantly, the flow cytometry panel included a CD4+CD25+FoxP3+ gate to identify classical regulatory T cells; no significant difference in this Treg-specific population was found between MAC and NOS-AC, indicating that the elevated CD4+CD25+ fraction is not driven by regulatory T-cell expansion and is more consistent with a dysfunctional activated effector compartment. Tumour cells in colorectal cancer models have been shown to drive CD4+ T cells towards a dysfunctional state [22,23,24] in which surface phenotype no longer reliably reflects effector function. CTLA-4 expression on tumour-infiltrating lymphocytes is dynamically regulated by T-cell receptor (TCR) signal strength: greater antigen stimulation drives more CTLA-4 to the immunological synapse [25]. Lower CTLA-4 in MAC would then reflect reduced antigen encounter, not a healthier T-cell response. This interpretation is consistent with both the HLA class II and mutational data: less antigen presentation, fewer neoantigens, less TCR stimulation and consequently less CTLA-4 induction.

The nominal upregulation of GMIP [19] and TNFRSF25 [26] in MAC points to two pathways that merit further investigation, though any interpretation must remain cautious given the sample size. GMIP is a RhoGAP-family regulator of RhoA activity that has been associated with immune-cell infiltration across tumour types [19], while TNFRSF25 (Death Receptor 3) is a TNF-superfamily death receptor whose signalling can trigger necroptotic cell death [26] and which has been described as a context-dependent biomarker and therapeutic target across cancers [27]. Whether their upregulation reflects a coordinated dysregulation of cytoskeletal and death receptor signalling in a microenvironment already defined by suppressed antigen presentation remains an open question; both genes are candidates for targeted validation in larger MAC cohorts.

The loss of MSH6 and PMS2 protein expression in the normal mucosa of MAC patients is another finding worth dwelling on. Epigenetic or post-translational silencing of MMR proteins in morphologically normal colorectal mucosa has been described before: field defects affecting PMS2 expression have been found in up to 70–95% of crypts within a 20 cm radius of CRC [28], through mechanisms that include promoter methylation and microRNA-mediated suppression [29,30,31]. Whether such field-level MMR instability is a driver of mucinous transformation, a consequence of it or a bystander phenomenon in genetically susceptible mucosa remains an open question. The PMS2 loss in healthy mucosa was also significant in MAC patients who received neoadjuvant therapy (28.6% vs. 0% in NOS-AC, *p* = 0.007) and may be potentially relevant: chemoradiotherapy itself is genotoxic and may unmask pre-existing epigenetic vulnerabilities. In the same patients, CD8+ infiltration in adjacent mucosa was higher after treatment in MAC than in NOS-AC, suggesting successful immune recruitment to the field that fails to translate into tumour regression, which is consistent with the well-documented resistance of MAC to neoadjuvant therapy [5,32].

The TCGA validation data are broadly supportive. No survival difference was seen between MAC and matched NOS-AC in the rectal cohort, replicating the PSM result. Tumour mutational burden was lower in MAC (*p* = 0.003). The transcriptomic signature was consistent with the mucinous phenotype (strong upregulation of MUC2, the TFF family and AGR2) and HIF1A overexpression in MAC points to a hypoxic microenvironment that may contribute independently to chemoresistance [33]. Lower VEGFA and PMS2 expression in MAC tumours suggest distinct angiogenic biology and continuing MMR protein instability at the tumour level. In the broader colorectal TCGA cohort, MAC showed higher expression of T-cell markers and immune checkpoints (PD-1, PD-L1, CTLA-4) than NOS-AC, which fits with a model in which MAC is immune-infiltrated but the infiltrate is dysfunctional. This interpretation is directly supported by the findings of Duggan et al. [10], who used multiplexed immunofluorescence to show that mucinous rectal cancer harbours a lymphocyte-rich, PD-1-enriched microenvironment regardless of MSI status.

Several limitations need to be acknowledged. The sample size is the most obvious: 16 MAC patients reflects the reality of studying a rare histotype in a cohort of 200 patients, and it means that many subgroup analyses (particularly the transcriptomics, feasible in only 4 MAC cases) are severely underpowered. The four MAC cases available for NanoString profiling were the only ones for which adequate snap-frozen tissue was available; the NOS-AC comparison group was selected based on stage at presentation to ensure comparability. Differential expression analysis applied Benjamini–Yekutieli (BY) correction for multiple testing across all 770 panel genes; no genes reached statistical significance after this correction. All transcriptomic *p*-values in the manuscript are therefore nominal, and these findings should be treated as exploratory biological signals pointing towards hypotheses that require validation in larger, adequately powered cohorts. Central, prospective MSI testing was not part of the original protocol, as most patients were enrolled before tumour MSI testing became routine in Italy. MSI data subsequently retrievable for a subset of patients (16 MAC and 100 NOS-AC) showed no significant difference between histotypes (MSI-high: 6.25% vs 8%, *p* = 0.808), which argues against microsatellite instability as a major driver of the observed immune differences; nonetheless, the incomplete coverage means a residual contribution cannot be entirely excluded. With regard to potential Lynch syndrome enrichment within the MAC subgroup, the median age at diagnosis was 59 years, which is not entirely suggestive of a hereditary syndrome and no excess of family history of colorectal or Lynch-associated cancers was observed in the MAC group; nonetheless, the absence of formal germline testing prevents definitive exclusion. Sporadic MSI-high arising from somatic MLH1 promoter hypermethylation accounts for the majority of MSI-high colorectal cancers [34], and neither MLH1 methylation testing nor a dedicated MSI assay was performed in our cohort. Such cases could therefore be present and undetected in either histological group, and we cannot exclude that they contribute to some of the immune differences we report. The two-antibody MMR screening approach is validated [16,17] but could miss isolated MLH1 or MSH2 loss. Furthermore, the loss of MSH6 and PMS2 protein expression observed in histologically normal mucosa was not validated by orthogonal methods such as methylation-specific PCR or a second antibody; technical artefacts contributing to this finding cannot therefore be excluded and molecular confirmation remains an important direction for future work. No prospective data on presenting symptoms were collected, and the haemoglobin difference, while statistically significant after adjustment, represents an indirect and imperfect surrogate for rectal bleeding; the interpretation that MAC bleeds less should be treated as a hypothesis requiring dedicated prospective confirmation. All biopsies of adjacent mucosa used for downstream analyses were confirmed by histopathological review to show preserved crypt architecture, absent high-grade dysplasia and absent active inflammation, following the same standard operating procedure applied in other IMMUNOREACT studies [35]. The retrospective design, even with PSM, cannot exclude residual confounding. The field cancerisation analyses are cross-sectional snapshots that do not fully explain how the immune microenvironment evolves in response to treatment. Finally, the TCGA validation cohort differs substantially from the IMMUNOREACT cohort in terms of neoadjuvant therapy exposure (fewer than 2% of TCGA cases received neoadjuvant treatment compared with 68.8% of MAC patients in our series), which limits direct biological comparability between the two datasets. Despite all of these limitations, the cross-cohort consistency gives us some confidence that the observations have a solid foundation.

## 5. Conclusions

The poor survival of rectal MAC patients appears, at least in part, to stem from a staging problem rather than from a fundamentally more malignant tumour. After adjusting for the considerable imbalance in stage at presentation, the histotype itself was not independently correlated to survival. The haemoglobin data may offer a biologically plausible reason for why these tumours present late: reduced rectal bleeding means a key alarm symptom is absent, and thus patients may not seek attention until obstruction or other late features develop. At the same time, MAC is not simply a late-stage NOS-AC. It has a distinct immune profile, visible even in adjacent normal tissue, with suppressed antigen presentation, low mutational burden and an immune infiltrate that is present in number but functionally compromised. Future work should focus on whether restoring antigen presentation in MAC is a viable therapeutic target and on defining the clinical features that should prompt clinicians to suspect mucinous histology earlier in the diagnostic pathway.

## Figures and Tables

**Table 1:** Patient characteristics in the general and PSM cohorts. Data are *n* (%) or median (IQR).

	General Cohort			PSM Cohort		
	MAC (*n* = 16)	NOS-AC (*n* = 184)	*p*	MAC (*n* = 16)	NOS-AC (*n* = 48)	*p*
Age, years	59 (12.5)	66 (21)	0.171	59 (12.5)	61 (23.3)	0.914
Male sex	11 (68.8%)	106 (57.6%)	0.195	11 (68.8%)	34 (70.8%)	0.875
Neoadjuvant therapy	11 (68.8%)	98 (53.3%)	0.233	11 (68.8%)	32 (66.7%)	0.878
T stage			0.006			0.189
T0	0 (0%)	39 (21.2%)		0 (0%)	1 (2.1%)	
Tis	0 (0%)	10 (5.4%)		0 (0%)	4 (8.3%)	
T1	0 (0%)	19 (10.3%)		0 (0%)	10 (20.8%)	
T2	4 (25.0%)	33 (17.9%)		4 (25.0%)	29 (60.4%)	
T3	8 (50.0%)	68 (37.0%)		8 (50.0%)	4 (8.3%)	
T4	4 (25.0%)	7 (3.8%)		4 (25.0%)	0 (0%)	
N stage			<0.001			0.224
N0	5 (31.3%)	136 (73.9%)		5 (31.3%)	21 (43.8%)	
N1	5 (31.3%)	28 (15.2%)		5 (31.3%)	17 (35.4%)	
N2	6 (37.5%)	12 (6.5%)		6 (37.5%)	10 (20.8%)	
M stage			0.039			0.394
M0	13 (81.3%)	169 (91.8%)		13 (81.3%)	43 (89.6%)	
M1	3 (18.8%)	7 (3.8%)		3 (18.8%)	5 (10.4%)	
Overall TNM stage			<0.001			0.433
Stage 0	0 (0%)	49 (26.6%)		0 (0%)	1 (2.1%)	
Stage I	3 (18.8%)	39 (21.2%)		3 (18.8%)	6 (12.5%)	
Stage II	1 (6.3%)	45 (25.0%)		1 (6.3%)	10 (20.8%)	
Stage III	9 (56.3%)	36 (20.0%)		9 (56.3%)	26 (54.2%)	
Stage IV	3 (18.8%)	7 (3.8%)		3 (18.8%)	5 (10.4%)	

In the overall cohort, MAC was associated with significantly worse OS (HR 2.53; 95% CI 1.03–6.23; *p* = 0.043) and DFS (HR 2.86; 95% CI 1.25–6.55; *p* = 0.013; Figure 2B). After PSM, these differences were no longer statistically significant (OS: HR 2.26; 95% CI 0.78–6.56; *p* = 0.134; DFS: HR 1.40; 95% CI 0.53–3.70; *p* = 0.493; Figure 2C), suggesting that the apparent survival disadvantage of MAC is largely attributable to advanced stage at presentation.

**Table 2:** Patient characteristics in the TCGA analysis. Data are *n* (%) or median (IQR).

	Total TCGA Colorectal Cohort			TCGA Rectal Cohort (PSM)		
	MAC (*n* = 61)	NOS-AC (*n* = 154)	*p*	MAC (*n* = 13)	NOS-AC (*n* = 65)	*p*
Age, years	67 (26)	65 (15)	0.369	59 (16)	62 (17)	0.571
Male sex	29 (47.5%)	81 (52.6%)	0.504	8 (61.5%)	35 (53.8%)	0.839
Neoadjuvant therapy	1 (1.6%)	1 (0.6%)	0.465	0 (0%)	0 (0%)	N/A
Overall TNM stage			0.701			0.730
Stage I	9 (14.8%)	29 (18.8%)		1 (7.7%)	9 (13.8%)	
Stage II	24 (39.3%)	50 (32.5%)		2 (15.4%)	19 (29.2%)	
Stage III	21 (34.4%)	47 (30.5%)		4 (30.8%)	26 (40.0%)	
Stage IV	7 (11.5%)	24 (15.6%)		2 (15.4%)	10 (15.4%)	

MAC demonstrated significantly lower tumour mutational burden (*p* = 0.003; Figure 5C) and higher expression of mucin-related genes (MUC2, *p* < 0.001; MUC6, *p* = 0.015; AGR2, *p* < 0.001; GALNT3, *p* < 0.001; ST6GALNAC1, *p* < 0.001), trefoil factors (TFF1, *p* = 0.002; TFF2, *p* = 0.018; TFF3, *p* < 0.001) and HIF1A (*p* = 0.002). Lower expression of PMS2 (*p* = 0.036) and VEGFA (*p* = 0.003) was also observed in MAC. In the broader colorectal TCGA cohort (61 MAC vs. 154 NOS-AC), MAC exhibited higher T-cell and immune checkpoint marker expression (PD-1, PD-L1, CTLA-4, Supplementary Figure S2), reinforcing the concept of an immune-infiltrated but functionally suppressed microenvironment.

## Data Availability

TCGA data are publicly available at https://www.cancer.gov/tcga (accessed on 1 November 2025). IMMUNOREACT cohort data are available from the corresponding author upon reasonable request, subject to data protection regulations and institutional approval.

## Supplementary Materials

The following supporting information can be downloaded at https://www.mdpi.com/article/10.3390/cancers18121943/s1. Table S1: Differential expression results (log2 fold change, nominal *p*-values and Benjamini–Yekutieli-adjusted *p*-values) for all 770 NanoString PanCancer IO 360™ panel genes in MAC vs. NOS-AC adjacent mucosa; Table S2: Frequency of mutated genes in the IMMUNOREACT 20 rectal cancer cohort; Figure S1: Clinical staging and flow cytometry analysis in the IMMUNOREACT 20 rectal cancer PSM cohort. (A) Clinical staging (in order: T stage, N stage, M stage, TNM stage) (B) Flow cytometry results for CD4+CD25+ T cells in neoplastic tissue. (C) Flow cytometry results for CD8+CD28+ T cells in neoplastic tissue after neoadjuvant therapy. MAC=Mucinous Adenocarcinoma. NOS: Not Otherwise Specified Adenocarcinoma (NOS-AC). PSM=Propensity Score Matched; Figure S2: TCGA whole colorectal cancer cohort analysis.
