# Supplementary material for: Ill Fate of Rectal Mucinous Adenocarcinoma: A Defect in Immunosurveillance or a Mucin Coating Effect?—The IMMUNOREACT 20 Study
Source: Cancers (Basel). 2026 Jun 15;18(12):1943. doi: 10.3390/cancers18121943 (PMC13297536; doi:10.3390/cancers18121943)

Supplementary Table S1. Differential expression results (log2 fold change, nominal p-values and Benjamini–Yekutieli-adjusted p-values) for all 770 NanoString PanCancer IO 360™ panel genes in MAC vs. NOS-AC adjacent mucosa.

Gene	Log2 fold change	P-value	BY.p.value
HLA-DQB1-mRNA	-3,97	0,00211	1
S100A12-mRNA	-3,29	0,00542	1
GMIP-mRNA	0,929	0,0072	1
S100A8-mRNA	-3,38	0,00919	1
RPL23-mRNA	-0,8	0,0101	1
HLA-DRB1-mRNA	-1,13	0,0125	1
TNFRSF25-mRNA	1,31	0,0168	1
HLA-DQA1-mRNA	-6,88	0,0176	1
UBE2T-mRNA	-0,88	0,018	1
S100A9-mRNA	-2,36	0,0185	1
HRAS-mRNA	0,815	0,0188	1
RPL7A-mRNA	-0,773	0,0191	1
FCGR1A-mRNA	-1,28	0,0205	1
C7-mRNA	-2,44	0,0216	1
UBA7-mRNA	0,748	0,0216	1
GPC4-mRNA	-0,741	0,0237	1
SMAD5-mRNA	-0,572	0,0247	1
PIAS4-mRNA	0,575	0,026	1
FGF13-mRNA	-2,06	0,0261	1
CDH2-mRNA	-1,43	0,0265	1
ZEB1-mRNA	-2,33	0,0266	1
CTLA4-mRNA	1,4	0,0273	1
SELE-mRNA	-2,56	0,0296	1
API5-mRNA	-0,425	0,0317	1
ITGB8-mRNA	-0,966	0,0318	1
FCN1-mRNA	-1,58	0,0329	1
ANGPT1-mRNA	-2,33	0,0329	1
PVRIG-mRNA	0,994	0,0332	1
CDKN1C-mRNA	-1,65	0,0335	1
IKBKB-mRNA	0,971	0,034	1
E2F3-mRNA	-0,513	0,0362	1
CHUK-mRNA	0,463	0,0362	1
AQP9-mRNA	-2,01	0,0374	1
LOXL2-mRNA	-1,18	0,0381	1
EDN1-mRNA	0,863	0,0412	1
PLA1A-mRNA	-1,32	0,0415	1
TGFBR1-mRNA	-1,34	0,0422	1
ZAP70-mRNA	1,23	0,0429	1
PRKAA2-mRNA	-2,06	0,0433	1
PFKM-mRNA	-1,44	0,044	1
NEIL1-mRNA	0,748	0,0453	1

CD47-mRNA	-0,51	0,0463	1
FSTL3-mRNA	-1,83	0,0464	1
IKBKG-mRNA	0,575	0,0469	1
KIT-mRNA	-1,3	0,0469	1
ICAM1-mRNA	-1,47	0,0479	1
NBN-mRNA	-0,548	0,0484	1
VEGFB-mRNA	-1,24	0,0488	1
STAT2-mRNA	0,38	0,0511	1
TLR8-mRNA	-1,14	0,0529	1
CCR2-mRNA	-0,753	0,0562	1
EIF4EBP1-mRNA	-0,501	0,0569	1
TGFBR2-mRNA	-0,988	0,0576	1
DLL1-mRNA	0,876	0,0581	1
TNFRSF14-mRNA	0,524	0,0594	1
CD247-mRNA	0,78	0,0603	1
SPP1-mRNA	-1,28	0,061	1
LDHB-mRNA	-0,824	0,0624	1
PTEN-mRNA	-0,722	0,0632	1
FCGR3A/B-mRNA	-1,23	0,0632	1
CXCL1-mRNA	-1,74	0,0636	1
IFNAR1-mRNA	-0,44	0,0648	1
ANGPT2-mRNA	-1,52	0,0653	1
IRF7-mRNA	0,685	0,0657	1
ITGA1-mRNA	-1,08	0,0668	1
C1QB-mRNA	-1	0,067	1
KLRK1-mRNA	0,597	0,0685	1
PKM-mRNA	-0,423	0,0685	1
PIK3CD-mRNA	0,854	0,0693	1
ANGPTL4-mRNA	-1,54	0,0714	1
DEPTOR-mRNA	-0,848	0,0727	1
VHL-mRNA	0,308	0,0746	1
NDUFA4L2-mRNA	-1,57	0,0747	1
ITGB3-mRNA	-1,72	0,0749	1
RB1-mRNA	-0,428	0,0754	1
PVR-mRNA	0,57	0,0755	1
BNIP3L-mRNA	-1	0,0777	1
CLEC14A-mRNA	-1,11	0,0778	1
MRC1-mRNA	-1,11	0,078	1
WNT5A-mRNA	0,634	0,0801	1
SNCA-mRNA	-1,35	0,0802	1
STAT3-mRNA	-0,543	0,0814	1
CXCL10-mRNA	-1,79	0,0823	1
PDCD1-mRNA	0,706	0,0824	1
A2M-mRNA	-1,2	0,0832	1
PDK1-mRNA	-0,558	0,084	1
LYZ-mRNA	0,484	0,0846	1
SIRPA-mRNA	-0,55	0,0855	1

TNFSF12-mRNA	-0,999	0,0857	1
IFI16-mRNA	-0,779	0,0873	1
PSMB5-mRNA	-0,45	0,0886	1
TAPBPL-mRNA	0,567	0,0912	1
HIF1A-mRNA	-0,653	0,0915	1
HMGB1-mRNA	-0,532	0,0931	1
CD96-mRNA	0,679	0,0942	1
CASP8-mRNA	0,413	0,0951	1
PIK3R1-mRNA	-0,884	0,0958	1
CD36-mRNA	-1,48	0,0961	1
LILRA5-mRNA	-1,1	0,097	1
PPARG-mRNA	0,566	0,0973	1
IDO1-mRNA	-1,29	0,0979	1
CCL8-mRNA	-1,49	0,0995	1
RPS6KB1-mRNA	-0,252	0,0999	1
PRKACB-mRNA	-0,783	0,1	1
ROBO4-mRNA	-1,52	0,102	1
SERPINH1-mRNA	-0,866	0,104	1
P2RY13-mRNA	-0,76	0,105	1
CX3CL1-mRNA	-1,12	0,105	1
APLNR-mRNA	1,37	0,106	1
MS4A4A-mRNA	-1,02	0,107	1
NLRC5-mRNA	0,698	0,107	1
IRF3-mRNA	0,628	0,109	1
FAS-mRNA	-0,438	0,11	1
PDCD1LG2-mRNA	-1,26	0,111	1
FLT1-mRNA	-1,05	0,112	1
IL1RN-mRNA	-0,958	0,112	1
PIK3CA-mRNA	-0,595	0,112	1
POLD1-mRNA	0,512	0,113	1
TPM1-mRNA	-1,1	0,115	1
TNKS-mRNA	-0,372	0,116	1
ATF3-mRNA	1,18	0,117	1
NOD2-mRNA	-0,545	0,119	1
LTBP1-mRNA	-1,13	0,124	1
HERC6-mRNA	0,671	0,125	1
PTGER4-mRNA	0,396	0,125	1
HDAC4-mRNA	-0,632	0,126	1
TSLP-mRNA	-0,827	0,127	1
RIPK2-mRNA	-0,501	0,131	1
LY96-mRNA	-0,767	0,131	1
PRR5-mRNA	0,527	0,134	1
ID4-mRNA	-1,37	0,135	1
CDH5-mRNA	-1,04	0,136	1
ITGA2-mRNA	-0,724	0,136	1
RPTOR-mRNA	0,416	0,137	1
BRD3-mRNA	0,313	0,138	1

RELA-mRNA	-0,301	0,138	1
TTC30A-mRNA	-0,437	0,139	1
TBXAS1-mRNA	-0,449	0,14	1
LTB-mRNA	0,877	0,14	1
GHR-mRNA	-0,782	0,141	1
CCL2-mRNA	-1,57	0,142	1
GBP1-mRNA	-1,01	0,144	1
PECAM1-mRNA	-0,901	0,145	1
BLK-mRNA	1,28	0,146	1
TRAF1-mRNA	0,577	0,146	1
ROCK1-mRNA	-0,944	0,146	1
NOTCH2-mRNA	-0,65	0,147	1
PGPEP1-mRNA	0,497	0,147	1
VCAM1-mRNA	-0,938	0,148	1
HK1-mRNA	-0,47	0,148	1
ITGAV-mRNA	-0,769	0,148	1
CXCL9-mRNA	-1,5	0,152	1
FGFR1-mRNA	-1,05	0,152	1
MGMT-mRNA	-0,371	0,152	1
TNFAIP6-mRNA	-1,24	0,154	1
KLRD1-mRNA	0,471	0,155	1
ELOB-mRNA	-0,375	0,157	1
CXCL11-mRNA	-1,68	0,158	1
MS4A6A-mRNA	-0,8	0,16	1
IL33-mRNA	-1,02	0,16	1
SMAP1-mRNA	0,244	0,161	1
ADORA2A-mRNA	-0,485	0,161	1
CDH11-mRNA	-0,669	0,164	1
ENTPD1-mRNA	-0,813	0,165	1
FANCA-mRNA	0,694	0,165	1
PIK3R5-mRNA	-0,573	0,166	1
CCL5-mRNA	0,593	0,166	1
LRRC32-mRNA	-1,08	0,167	1
MICB-mRNA	-0,557	0,168	1
MAPK10-mRNA	-0,781	0,168	1
NFKB1-mRNA	-0,169	0,17	1
BAX-mRNA	0,27	0,172	1
NCAM1-mRNA	-0,683	0,173	1
NFKBIE-mRNA	-0,331	0,176	1
ERBB2-mRNA	0,46	0,176	1
PPARGC1B-mRNA	0,713	0,176	1
DNMT1-mRNA	0,409	0,177	1
BRD4-mRNA	0,233	0,178	1
ITGAL-mRNA	0,549	0,18	1
CRABP2-mRNA	-0,799	0,182	1
CD69-mRNA	0,646	0,184	1
CD44-mRNA	-0,474	0,185	1

COL6A3-mRNA	-0,808	0,186	1
SLC1A5-mRNA	-0,498	0,19	1
MFGE8-mRNA	-1,15	0,191	1
SRP54-mRNA	-0,227	0,191	1
CPA3-mRNA	-0,927	0,191	1
LILRB2-mRNA	-0,569	0,195	1
CD19-mRNA	0,982	0,195	1
FPR1-mRNA	-1,19	0,197	1
GLS-mRNA	0,363	0,197	1
ARNT2-mRNA	-0,587	0,199	1
GOT2-mRNA	-0,416	0,204	1
CD3G-mRNA	0,776	0,204	1
CD79B-mRNA	1,24	0,206	1
ITGB2-mRNA	-0,589	0,207	1
HLA-C-mRNA	0,356	0,209	1
SFXN1-mRNA	0,373	0,21	1
EGR1-mRNA	1,03	0,211	1
FCGR2B-mRNA	-0,683	0,211	1
CD209-mRNA	-0,781	0,211	1
HLA-DPA1-mRNA	-0,694	0,212	1
RAD51-mRNA	-0,636	0,212	1
EPM2AIP1-mRNA	-0,315	0,214	1
DTX4-mRNA	0,559	0,216	1
AXIN1-mRNA	0,284	0,219	1
CCND3-mRNA	0,204	0,22	1
TWF1-mRNA	-0,232	0,22	1
IFITM1-mRNA	-0,641	0,221	1
MTOR-mRNA	0,238	0,223	1
JAG1-mRNA	-0,496	0,224	1
PC-mRNA	0,44	0,225	1
PDZK1IP1-mRNA	0,818	0,226	1
ZEB2-mRNA	-0,618	0,227	1
OLFML2B-mRNA	-0,856	0,228	1
SOX10-mRNA	-0,622	0,229	1
HSD11B1-mRNA	-0,787	0,229	1
FUT4-mRNA	0,329	0,23	1
HLA-DPB1-mRNA	-0,511	0,23	1
HLA-F-mRNA	0,484	0,231	1
MMP1-mRNA	-1	0,232	1
KDR-mRNA	-0,557	0,233	1
GLUL-mRNA	-0,372	0,234	1
TAF3-mRNA	-0,311	0,234	1
EGF-mRNA	-0,416	0,234	1
CSF1R-mRNA	-0,624	0,237	1
TNFRSF10B-mRNA	0,368	0,241	1
ZC3H12A-mRNA	0,536	0,241	1

SAMSN1-mRNA	-0,623	0,242	1
SELP-mRNA	-0,76	0,242	1
BATF3-mRNA	-0,609	0,244	1
RIPK3-mRNA	0,616	0,245	1
FLNB-mRNA	0,303	0,247	1
SLC7A5-mRNA	-0,587	0,247	1
RASAL1-mRNA	0,736	0,247	1
TNFRSF1B-mRNA	0,364	0,247	1
RBL2-mRNA	-0,234	0,248	1
OAS1-mRNA	0,628	0,251	1
H2AFX-mRNA	-0,562	0,252	1
NCR1-mRNA	0,336	0,253	1
MMRN2-mRNA	-0,748	0,253	1
ICAM3-mRNA	-0,247	0,254	1
TIGIT-mRNA	0,608	0,255	1
PDGFA-mRNA	0,311	0,255	1
ITGAM-mRNA	-0,481	0,256	1
FCGR2A-mRNA	-0,555	0,257	1
CTNNB1-mRNA	-0,205	0,261	1
CCND2-mRNA	-0,603	0,261	1
CD7-mRNA	0,432	0,262	1
MYCT1-mRNA	-0,6	0,263	1
LILRA1-mRNA	-0,617	0,263	1
IFNGR2-mRNA	0,28	0,267	1
MB21D1-mRNA	-0,263	0,267	1
CXCL3-mRNA	0,739	0,268	1
GLI1-mRNA	-0,558	0,27	1
AKT1-mRNA	-0,132	0,275	1
NOTCH1-mRNA	0,263	0,276	1
LGALS9-mRNA	0,477	0,276	1
MYC-mRNA	-0,538	0,276	1
C1QA-mRNA	-0,475	0,277	1
CD74-mRNA	-0,351	0,277	1
TLR2-mRNA	-0,628	0,278	1
CD163-mRNA	-0,797	0,278	1
FCGRT-mRNA	0,297	0,279	1
LDHA-mRNA	-0,367	0,279	1
TLR7-mRNA	-0,546	0,281	1
GBP4-mRNA	-0,771	0,283	1
NT5E-mRNA	0,421	0,286	1
ERO1A-mRNA	-0,37	0,286	1
HELLS-mRNA	0,479	0,286	1
IFI35-mRNA	0,312	0,287	1
DDB2-mRNA	0,371	0,288	1
CCL4-mRNA	0,568	0,291	1
SNAI1-mRNA	-0,54	0,292	1
CDKN2A-mRNA	-0,367	0,292	1

IL2RB-mRNA	0,465	0,293	1
LAIR1-mRNA	-0,389	0,296	1
CD276-mRNA	-0,171	0,297	1
GIMAP4-mRNA	-0,472	0,299	1
MSH2-mRNA	-0,307	0,299	1
FPR3-mRNA	-0,477	0,301	1
HMGA1-mRNA	-0,441	0,302	1
VCAN-mRNA	-0,57	0,305	1
TMEM140-mRNA	0,525	0,305	1
PIK3R2-mRNA	0,254	0,306	1
TLR5-mRNA	-0,356	0,307	1
NKG7-mRNA	0,39	0,308	1
APOL6-mRNA	0,408	0,311	1
KAT2B-mRNA	0,185	0,312	1
TGFB2-mRNA	-1,35	0,314	1
THBD-mRNA	-0,832	0,315	1
AXL-mRNA	-0,61	0,316	1
PRKCA-mRNA	0,293	0,319	1
CXCL13-mRNA	1,05	0,319	1
CSF3R-mRNA	-0,762	0,32	1
TGFB3-mRNA	-0,984	0,321	1
CTSW-mRNA	0,438	0,323	1
MAP3K12-mRNA	0,55	0,324	1
TPSAB1/B2-mRNA	-0,64	0,326	1
TP53-mRNA	-0,267	0,326	1
HNF1A-mRNA	1,18	0,326	1
FOSL1-mRNA	-1,14	0,327	1
ENO1-mRNA	-0,368	0,327	1
CD2-mRNA	0,44	0,327	1
BCAT1-mRNA	-0,488	0,328	1
MSH6-mRNA	-0,334	0,33	1
ARID1A-mRNA	-0,202	0,33	1
SLC16A1-mRNA	0,553	0,33	1
EZH2-mRNA	0,3	0,331	1
PLA2G2A-mRNA	-0,757	0,331	1
ROR2-mRNA	-0,603	0,331	1
THY1-mRNA	-0,598	0,333	1
P4HA2-mRNA	-0,32	0,335	1
THBS1-mRNA	-0,509	0,336	1
CDKN2B-mRNA	0,605	0,337	1
MS4A1-mRNA	0,968	0,339	1
MICA-mRNA	0,291	0,342	1
DLL4-mRNA	0,381	0,344	1
MMP7-mRNA	-0,769	0,346	1
IRF4-mRNA	0,673	0,347	1
FAM30A-mRNA	0,866	0,348	1

GLUD1-mRNA	0,0829	0,349	1
ALDOA-mRNA	-0,146	0,35	1
IHH-mRNA	0,722	0,35	1
TCF3-mRNA	0,15	0,351	1
COL17A1-mRNA	0,633	0,352	1
TLR1-mRNA	-0,238	0,353	1
NFIL3-mRNA	-0,591	0,354	1
PTCD2-mRNA	-0,213	0,36	1
WDR76-mRNA	-0,433	0,361	1
OAS3-mRNA	0,372	0,361	1
HAVCR2-mRNA	-0,349	0,363	1
MS4A2-mRNA	-0,462	0,363	1
VEGFA-mRNA	-0,779	0,363	1
PFKFB3-mRNA	-0,417	0,365	1
RICTOR-mRNA	-0,229	0,365	1
BRCA1-mRNA	-0,357	0,366	1
IFIT1-mRNA	0,526	0,367	1
SGK1-mRNA	0,595	0,37	1
COL5A1-mRNA	-0,501	0,371	1
CD40-mRNA	-0,351	0,372	1
TAP1-mRNA	-0,414	0,375	1
IRF9-mRNA	0,258	0,377	1
EGFR-mRNA	-0,207	0,378	1
CEBPB-mRNA	-0,491	0,379	1
BCL2-mRNA	-0,161	0,379	1
PDGFB-mRNA	-0,344	0,379	1
CD45RO-mRNA	-0,346	0,379	1
IRF8-mRNA	0,353	0,379	1
NGFR-mRNA	-0,411	0,381	1
GZMA-mRNA	0,675	0,381	1
SAMD9-mRNA	0,459	0,382	1
LCK-mRNA	0,585	0,383	1
CD86-mRNA	-1,1	0,388	1
KRAS-mRNA	-0,198	0,392	1
P4HA1-mRNA	-0,284	0,395	1
HLA-DMB-mRNA	-0,35	0,401	1
PMS2-mRNA	0,16	0,401	1
HDC-mRNA	-0,427	0,401	1
BID-mRNA	-0,329	0,403	1
HLA-B-mRNA	0,322	0,403	1
WNT5B-mRNA	0,421	0,405	1
LIF-mRNA	-0,415	0,411	1
SLC11A1-mRNA	-0,657	0,412	1
CCNB1-mRNA	-0,462	0,412	1
CXCL12-mRNA	0,387	0,413	1
CD3D-mRNA	0,55	0,415	1
CDC20-mRNA	-0,525	0,417	1

NFATC2-mRNA	-0,423	0,417	1
TNF-mRNA	0,402	0,417	1
RELN-mRNA	-0,463	0,418	1
IFI27-mRNA	0,547	0,42	1
CLEC7A-mRNA	-0,355	0,42	1
BRCA2-mRNA	-0,231	0,422	1
BCL6B-mRNA	-0,468	0,423	1
FCRL2-mRNA	0,528	0,425	1
DPP4-mRNA	0,442	0,429	1
IFITM2-mRNA	-0,415	0,431	1
CCL18-mRNA	-0,484	0,432	1
LAG3-mRNA	0,439	0,435	1
RIPK1-mRNA	0,122	0,436	1
HLA-DRA-mRNA	-0,321	0,437	1
CD45RA-mRNA	0,651	0,437	1
MAP3K7-mRNA	0,0903	0,439	1
MARCO-mRNA	-0,717	0,44	1
CD8A-mRNA	0,384	0,44	1
ICAM2-mRNA	-0,314	0,441	1
STAT1-mRNA	-0,377	0,441	1
PDGFRB-mRNA	-0,54	0,442	1
IL18R1-mRNA	-0,376	0,442	1
CEP55-mRNA	0,359	0,443	1
CBLC-mRNA	0,919	0,444	1
TNFSF9-mRNA	0,39	0,456	1
MET-mRNA	-0,275	0,456	1
TMEM173-mRNA	-0,188	0,456	1
TNFRSF17-mRNA	-0,667	0,459	1
IL6R-mRNA	0,164	0,461	1
XCL1/2-mRNA	0,36	0,467	1
MAP3K5-mRNA	0,117	0,471	1
CCNA1-mRNA	-0,398	0,471	1
PF4-mRNA	-0,43	0,472	1
GPSM3-mRNA	-0,316	0,472	1
PROM1-mRNA	0,422	0,472	1
EIF2AK2-mRNA	-0,178	0,477	1
TNFRSF9-mRNA	0,351	0,479	1
CES3-mRNA	1,18	0,48	1
SHC2-mRNA	0,281	0,482	1
MAP3K8-mRNA	-0,354	0,486	1
TNFSF13-mRNA	0,247	0,489	1
DUSP1-mRNA	0,518	0,49	1
PIK3CG-mRNA	-0,285	0,492	1
MAML2-mRNA	-0,169	0,497	1
GBP2-mRNA	-0,354	0,497	1
JAK3-mRNA	0,367	0,497	1
TNFSF10-mRNA	0,431	0,497	1

NFKBIA-mRNA	-0,319	0,501	1
PARP12-mRNA	0,324	0,503	1
HES1-mRNA	0,136	0,503	1
FZD8-mRNA	-0,148	0,504	1
ITPK1-mRNA	-0,0845	0,506	1
SPIB-mRNA	0,426	0,508	1
MX1-mRNA	0,419	0,51	1
APC-mRNA	0,094	0,512	1
BBC3-mRNA	0,222	0,516	1
CD300A-mRNA	-0,244	0,516	1
CTSS-mRNA	-0,296	0,516	1
WNT4-mRNA	0,311	0,517	1
CCR5-mRNA	0,278	0,519	1
IL22RA1-mRNA	0,4	0,52	1
HLA-DMA-mRNA	0,188	0,521	1
MAGEB2-mRNA	0,338	0,523	1
CD274-mRNA	0,309	0,523	1
CXCL14-mRNA	-0,343	0,525	1
CLECL1-mRNA	0,38	0,526	1
IL32-mRNA	0,388	0,528	1
CD8B-mRNA	0,332	0,53	1
CD4-mRNA	-0,328	0,53	1
TLR3-mRNA	0,375	0,531	1
RASGRF1-mRNA	0,457	0,533	1
UBE2C-mRNA	-0,374	0,535	1
CD3E-mRNA	0,286	0,538	1
RUNX3-mRNA	0,413	0,538	1
CASP1-mRNA	-0,312	0,539	1
JAK2-mRNA	-0,367	0,539	1
SELL-mRNA	-0,501	0,54	1
PALMD-mRNA	-0,395	0,541	1
CSF1-mRNA	-0,238	0,542	1
BIRC5-mRNA	-0,329	0,543	1
TNFSF8-mRNA	0,352	0,547	1
B2M-mRNA	0,195	0,549	1
OASL-mRNA	0,422	0,553	1
CMKLR1-mRNA	-0,28	0,554	1
SIGLEC1-mRNA	0,347	0,554	1
RAD51C-mRNA	-0,105	0,555	1
IL1B-mRNA	-0,51	0,556	1
ITGAX-mRNA	0,277	0,557	1
IFI6-mRNA	-0,365	0,558	1
TICAM1-mRNA	0,188	0,56	1
CD28-mRNA	0,338	0,563	1
TCL1A-mRNA	1,13	0,564	1
SOX11-mRNA	-0,385	0,564	1
IL11RA-mRNA	0,31	0,564	1

NOS2-mRNA	-0,612	0,566	1
ITGA6-mRNA	-0,213	0,567	1
CD80-mRNA	0,284	0,567	1
CASP9-mRNA	0,142	0,568	1
SIRPB2-mRNA	-0,27	0,57	1
BAD-mRNA	0,119	0,572	1
BIRC3-mRNA	-0,326	0,572	1
SERPINA1-mRNA	0,238	0,575	1
TREM2-mRNA	-0,24	0,58	1
WNT2B-mRNA	0,274	0,581	1
EIF2B4-mRNA	0,111	0,582	1
RORC-mRNA	0,669	0,584	1
MKI67-mRNA	0,304	0,584	1
HEY1-mRNA	-0,151	0,588	1
BAMBI-mRNA	0,349	0,59	1
TYMP-mRNA	-0,291	0,592	1
CD84-mRNA	0,263	0,592	1
JAK1-mRNA	0,0574	0,594	1
CCL14-mRNA	-0,242	0,596	1
TNFRSF11A-mRNA	0,449	0,6	1
CD70-mRNA	0,263	0,602	1
LY9-mRNA	-0,341	0,603	1
TRAT1-mRNA	0,379	0,604	1
SYK-mRNA	0,147	0,605	1
NECTIN2-mRNA	0,0946	0,607	1
LAMB3-mRNA	0,29	0,609	1
OAS2-mRNA	-0,241	0,615	1
CXCL16-mRNA	0,175	0,617	1
CXCL8-mRNA	0,781	0,618	1
RSAD2-mRNA	0,382	0,618	1
MELK-mRNA	-0,329	0,625	1
ADM-mRNA	-0,285	0,628	1
GNLY-mRNA	-0,208	0,628	1
MFNG-mRNA	0,239	0,631	1
IER3-mRNA	0,329	0,632	1
ESR1-mRNA	-0,347	0,633	1
CEACAM3-mRNA	0,179	0,635	1
NECTIN1-mRNA	0,159	0,637	1
IL2RA-mRNA	-0,377	0,639	1
PSMB9-mRNA	-0,246	0,643	1
TAP2-mRNA	0,193	0,65	1
DAB2-mRNA	-0,141	0,65	1
CXorf36-mRNA	-0,287	0,658	1
HCK-mRNA	-0,137	0,66	1
EPCAM-mRNA	0,547	0,663	1
CD40LG-mRNA	0,311	0,671	1

PTPRC-mRNA	-0,208	0,672	1
IL2RG-mRNA	0,21	0,674	1
SLAMF7-mRNA	-0,296	0,676	1
VEGFC-mRNA	-0,196	0,678	1
CD79A-mRNA	0,314	0,681	1
BTLA-mRNA	-0,358	0,685	1
NID2-mRNA	-0,149	0,688	1
FADD-mRNA	0,101	0,692	1
FAM124B-mRNA	-0,174	0,693	1
IRF5-mRNA	0,1	0,694	1
NFKB2-mRNA	0,131	0,702	1
CSF2RB-mRNA	0,173	0,702	1
CCND1-mRNA	-0,101	0,711	1
ITGA4-mRNA	0,146	0,716	1
HLA-E-mRNA	0,0835	0,716	1
CD45RB-mRNA	-0,127	0,718	1
TPI1-mRNA	-0,0738	0,718	1
SFRP1-mRNA	-0,253	0,719	1
CDH1-mRNA	0,246	0,72	1
IFNGR1-mRNA	0,0653	0,724	1
BMP2-mRNA	-0,204	0,724	1
IL10RA-mRNA	0,152	0,726	1
LAMA1-mRNA	0,317	0,73	1
IRF2-mRNA	0,0806	0,73	1
DUSP5-mRNA	-0,202	0,731	1
LAMC2-mRNA	-0,205	0,734	1
CCNE1-mRNA	0,137	0,737	1
TGFB1-mRNA	-0,165	0,74	1
GOT1-mRNA	-0,099	0,74	1
CCR4-mRNA	0,226	0,746	1
KIF2C-mRNA	-0,162	0,747	1
CXCR3-mRNA	0,124	0,747	1
CXCL5-mRNA	-0,534	0,752	1
RRM2-mRNA	0,176	0,754	1
PSMB8-mRNA	-0,162	0,758	1
MYD88-mRNA	0,0822	0,762	1
MRE11-mRNA	-0,0669	0,762	1
EXO1-mRNA	-0,172	0,763	1
TNFSF4-mRNA	-0,0857	0,765	1
ICOS-mRNA	0,25	0,766	1
CD5-mRNA	0,144	0,767	1
GIMAP6-mRNA	-0,144	0,767	1
SBNO2-mRNA	0,0956	0,768	1
TNFAIP3-mRNA	-0,18	0,768	1
BLM-mRNA	-0,129	0,77	1
TLR9-mRNA	0,133	0,77	1
ACVR1C-mRNA	0,11	0,77	1

ARG2-mRNA	-0,221	0,771	1
NF1-mRNA	0,0556	0,773	1
GZMK-mRNA	0,117	0,773	1
ICOSLG-mRNA	0,0965	0,775	1
ISG15-mRNA	0,198	0,776	1
AREG-mRNA	0,225	0,777	1
ULBP2-mRNA	-0,149	0,778	1
BBS1-mRNA	-0,0731	0,779	1
PTGS2-mRNA	-0,266	0,779	1
IL15-mRNA	0,0934	0,781	1
HDAC5-mRNA	0,0941	0,781	1
MXI1-mRNA	0,138	0,785	1
TYMS-mRNA	0,0941	0,793	1
BCL2L1-mRNA	0,0409	0,798	1
RELB-mRNA	0,0868	0,799	1
PTPN11-mRNA	-0,0698	0,803	1
PSMB10-mRNA	-0,0909	0,809	1
SH2D1A-mRNA	0,111	0,811	1
CDK2-mRNA	-0,0524	0,813	1
DUSP2-mRNA	0,162	0,814	1
IL16-mRNA	0,114	0,814	1
TRIM21-mRNA	0,0574	0,814	1
ITGAE-mRNA	-0,0424	0,817	1
HLA-A-mRNA	0,19	0,819	1
FBP1-mRNA	0,0776	0,823	1
ALDOC-mRNA	-0,125	0,824	1
CD48-mRNA	-0,127	0,826	1
SOCS1-mRNA	0,103	0,836	1
CD27-mRNA	0,169	0,838	1
HLA-DOB-mRNA	0,117	0,844	1
PCK2-mRNA	0,075	0,844	1
CCL20-mRNA	-0,143	0,849	1
TNFSF13B-mRNA	-0,0735	0,851	1
MLH1-mRNA	-0,0279	0,852	1
RAD50-mRNA	-0,0379	0,855	1
VSIR-mRNA	0,0381	0,859	1
RNLS-mRNA	-0,0302	0,863	1
GPR160-mRNA	-0,0896	0,864	1
SERPINB5-mRNA	0,164	0,864	1
CD38-mRNA	0,0736	0,868	1
PRF1-mRNA	-0,0711	0,875	1
CDK6-mRNA	-0,038	0,876	1
MMP9-mRNA	0,0939	0,882	1
IL12RB2-mRNA	0,0589	0,882	1
CD58-mRNA	-0,0184	0,883	1
LILRB4-mRNA	0,0668	0,883	1
IGF2R-mRNA	-0,0289	0,884	1

CXCR6-mRNA	0,0887	0,886	1
F2RL1-mRNA	0,096	0,89	1
FYN-mRNA	-0,0626	0,891	1
DTX3L-mRNA	0,0444	0,892	1
C5-mRNA	-0,0398	0,895	1
IL6-mRNA	0,203	0,895	1
NRAS-mRNA	-0,0199	0,895	1
IL1R2-mRNA	-0,0368	0,897	1
PLOD2-mRNA	-0,0669	0,902	1
C5AR1-mRNA	0,0957	0,904	1
IFIT2-mRNA	0,0555	0,906	1
ATM-mRNA	0,0417	0,906	1
CCL22-mRNA	0,112	0,906	1
TDO2-mRNA	-0,0832	0,906	1
SPRY4-mRNA	-0,0453	0,907	1
APOE-mRNA	0,0554	0,908	1
IL7R-mRNA	-0,0799	0,909	1
HDAC11-mRNA	0,0173	0,91	1
CXCR4-mRNA	0,076	0,91	1
IFIT3-mRNA	0,0675	0,911	1
BRIP1-mRNA	-0,045	0,912	1
SREBF1-mRNA	-0,0284	0,913	1
CD14-mRNA	0,0468	0,913	1
CCL21-mRNA	-0,079	0,913	1
PRLR-mRNA	-0,0465	0,917	1
CXCL2-mRNA	-0,0756	0,925	1
HDAC3-mRNA	-0,00874	0,929	1
IL18-mRNA	-0,0397	0,931	1
COL4A5-mRNA	-0,0575	0,932	1
STAT4-mRNA	0,039	0,934	1
CYBB-mRNA	-0,0345	0,935	1
HK2-mRNA	0,0358	0,938	1
TNFRSF10D-mRNA	0,0253	0,939	1
SLC2A1-mRNA	0,0147	0,943	1
CD1C-mRNA	-0,0457	0,946	1
IRF1-mRNA	0,0269	0,947	1
BNIP3-mRNA	-0,0135	0,958	1
TNFRSF18-mRNA	-0,0235	0,963	1
PARP9-mRNA	-0,0247	0,964	1
CENPF-mRNA	-0,0288	0,965	1
TNFRSF11B-mRNA	-0,0139	0,967	1
ADAM12-mRNA	-0,0122	0,969	1
TAPBP-mRNA	-0,00822	0,973	1
KLRB1-mRNA	0,0122	0,978	1
CCL19-mRNA	-0,0208	0,979	1

CCL3/L1-mRNA	-0,0209	0,979	1
TNFRSF1A-mRNA	0,00436	0,979	1
TIE1-mRNA	0,0134	0,98	1
PARP4-mRNA	0,00552	0,985	1
CD68-mRNA	-0,00567	0,985	1
IFIH1-mRNA	-0,00579	0,987	1
WNT11-mRNA	-0,0106	0,988	1
TLR4-mRNA	0,00484	0,988	1
IL2-mRNA	-0,00552	0,992	1
CDKN1A-mRNA	-0,00314	0,994	1
ANLN-mRNA	0,00192	0,996	1
CASP3-mRNA	0,00161	0,996	1
C2-mRNA	2,81E-15	1	1

Supplementary Table S2. Frequency of mutated genes in the IMMUNOREACT 20 rectal cancer cohort.

	MAC	NOS-AC	p value
AKT1	0/2 (0%)	24/61 (39.3%)	0.379
APC	0/2 (0%)	33/61 (54.1%)	0.223
ATM	1/2 (50%)	30/61 (49.2%)	0.762
CCNE1	0/2 (0%)	7/61 (11.5%)	1
CDKN2a	0/2 (0%)	10/61 (16.4%)	1
CSF1r	0/2 (0%)	3/61 (4.9%)	1
CTNNB1	0/2 (0%)	16/61 (26.2%)	0.554
DDR2	0/2 (0%)	26/61 (42.6%)	0.341
ERB B2	2/2 (100%)	43/61 (70.5%)	1
FBXW7	1/2 (50%)	42/61 (68.9%)	0.538
FGFR1	1/2 (50%)	17/61 (27.9%)	0.922
IDH1	0/2 (0%)	7/61 (11.5%)	0.789
IDH2	0/2 (0%)	4/61 (6.6%)	1
KDR	0/2 (0%)	21/61 (34.4%)	0.441
KIT	1/2 (50%)	23/61 (37.7%)	0.859
KRAS	0/2 (0%)	15/61 (24.6%)	1
MET	0/2 (0%)	21/61 (34.4%)	0.441
NOTCH1	0/2 (0%)	26/61 (42.6%)	0.341
PIK3ca	0/2 (0%)	18/61 (29.5%)	0.507
PIK3r1	0/2 (0%)	24/61 (39.3%)	1
pTEN	0/2 (0%)	27/61 (44.3%)	0.323
mut RB1	0/2 (0%)	14/61 (23%)	0.602
SMAD4	0/2 (0%)	22/61 (36.1%)	0.42
STK11	0/2 (0%)	13/61 (21.3%)	0.627
tP53	0/2 (0%)	17/61 (27.9%)	0.53
VHL	1/2 (50%)	9/61 (14.8%)	0.294
mut CDH1	0/2 (0%)	26/61 (42.6%)	0.341
mut ALK	0/2 (0%)	8/61 (13.1%)	1
mut MLH1	0/2 (0%)	2/60 (3.3%)	1
mut ABL1	0/2 (0%)	1/61 (1.6%)	1

mut EGFR	0/2 (0%)	18/61 (29.5%)	0.507
mut ERB B3	0/2 (0%)	17/61 (27.9%)	0.53
mut FGFR3	0/2 (0%)	11/61 (18%)	1
mut GNAQ	0/2 (0%)	5/61 (8.2%)	1
mut ERB B2 (2)	0/2 (0%)	15/61 (24.6%)	1
mut NRAS	0/2 (0%)	4/61 6.6%)	1
mut H3F3a	0/2 (0%)	7/61 (11.5%)	1
mut GNA11	0/2 (0%)	4/60 (6.7%)	1
mut PTPN11	0/2 (0%)	6/61 (9.8%)	1
mut hRAS	0/2 (0%)	5/61 (8.2%)	1
mut SMO	0/2 (0%)	5/61 (8.2%)	1
mut JAK3	0/2 (0%)	1/61 (1.6%)	1
mut SMARCB1	0/2 (0%)	7/61 (11.5%)	1
mut FIT3	1/2 (50%)	9/61 (14.8%)	0.294
mut BRAF	0/2 (0%)	14/61 (23%)	0.602
mut GNAS	0/2 (0%)	3/61 (4.9%)	1
mut MAP2K1	0/2 (0%)	5/61 (8.2%)	1
mut FOXL2	0/2 (0%)	4/61 (6.6%)	1
mut ROS1	0/2 (0%)	12/61 (19.7%)	0.653
mut RET	0/2 (0%)	8/61 (13.1%)	0.76
mut PDGFRA	0/2 (0%)	11/61 (18%)	0.679
mut JAK2	0/2 (0%)	3/61 (4.9%)	1
mut ESR1	0/2 (0%)	6/61 (9.8%)	1
mut RHOA	0/2 (0%)	4/61 (6.6%)	1
mut FGFR2	0/2 (0%)	2/61 (3.3%)	1
mut MDM2	0/2 (0%)	1/61 (1.6%)	1
mut CDK4	0/2 (0%)	2/61 (3.3%)	1

Supplementary Figure S1. Clinical staging and flow cytometry analysis in the IMMUNOREACT 20 rectal cancer PSM cohort. (A) Clinical staging (in order: T stage, N stage, M stage, TNM stage) (B) Flow cytometry results for CD4+CD25+ T cells in neoplastic tissue. (C) Flow cytometry results for CD8+CD28+ T cells in neoplastic tissue after neoadjuvant therapy. MAC=Mucinous Adenocarcinoma. NOS: Not Otherwise Specified Adenocarcinoma (NOS-AC). PSM=Propensity Score Matched.

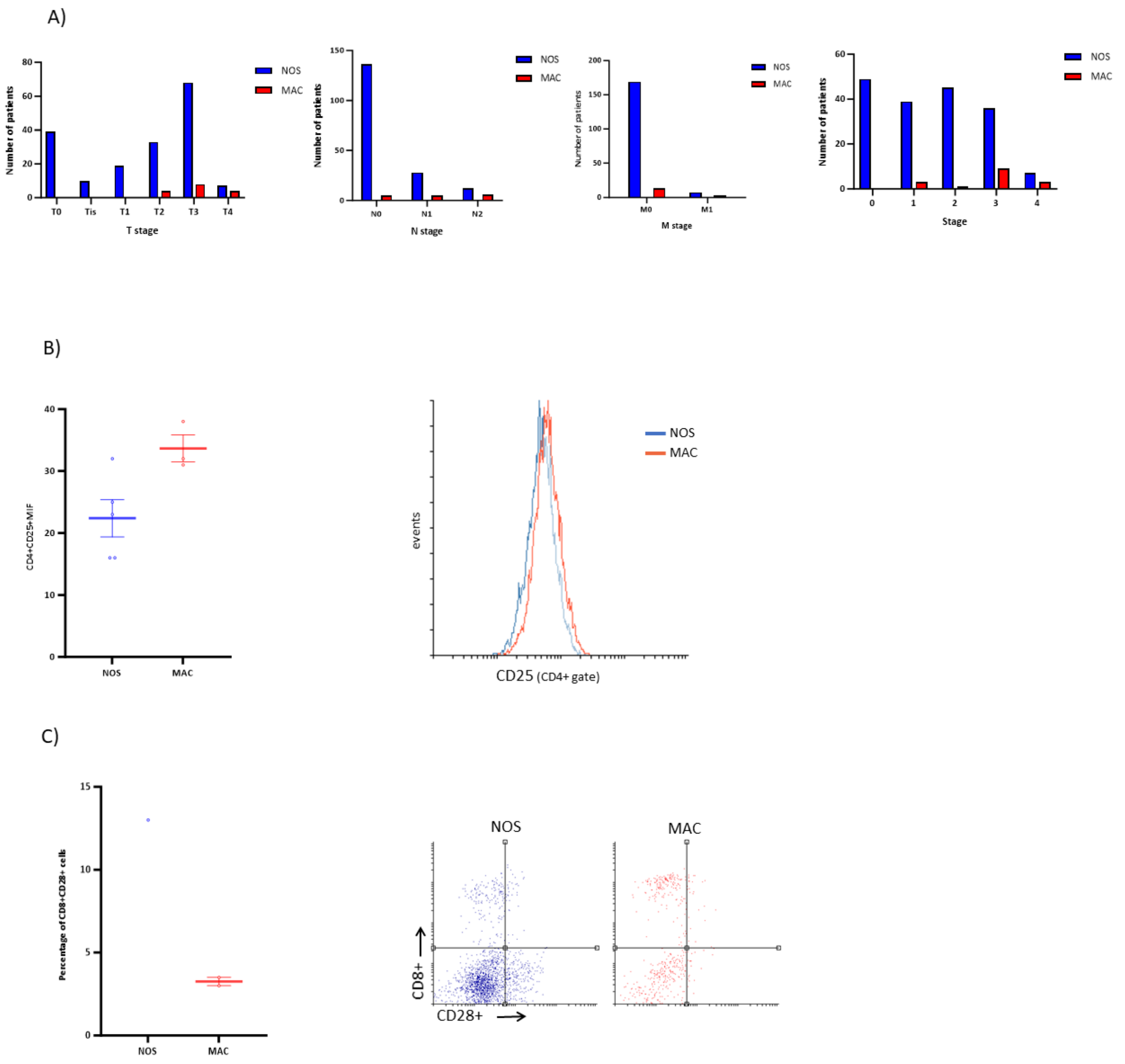

Supplementary Figure S2. TCGA whole colorectal cancer cohort analysis.

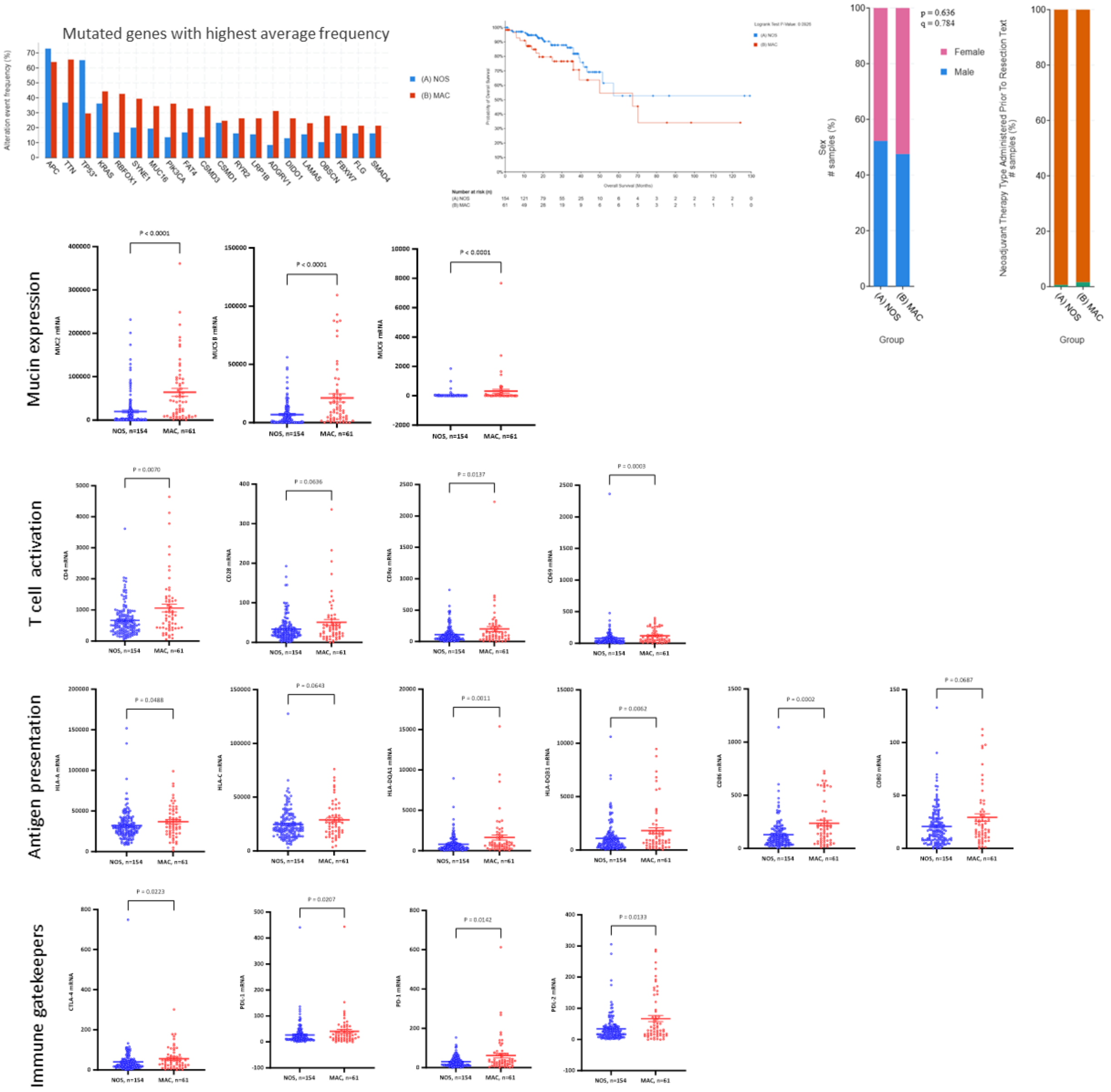

Supplement: Supplementary file 1 [file cancers-18-01943-s001.zip › cancers-4344292-supplementary.pdf]
